# Avoiding Pitfalls of Internal Controls: Validation of Reference Genes for Analysis by qRT-PCR and Western Blot throughout Rat Retinal Development

**DOI:** 10.1371/journal.pone.0043028

**Published:** 2012-08-20

**Authors:** Maurício Rocha-Martins, Brian Njaine, Mariana S. Silveira

**Affiliations:** 1 Neurobiology Department, Instituto de Biofísica Carlos Chagas Filho, Universidade Federal do Rio de Janeiro, Rio de Janeiro, Brazil; 2 Cell Therapy and Bioengeneering Department, Instituto de Biofísica Carlos Chagas Filho, Universidade Federal do Rio de Janeiro, Rio de Janeiro, Brazil; Federal University of Rio de Janeiro, Brazil

## Abstract

**Background:**

Housekeeping genes have been commonly used as reference to normalize gene expression and protein content data because of its presumed constitutive expression. In this paper, we challenge the consensual idea that housekeeping genes are reliable controls for expression studies in the retina through the investigation of a panel of reference genes potentially suitable for analysis of different stages of retinal development.

**Methodology/Principal Findings:**

We applied statistical tools on combinations of retinal developmental stages to assess the most stable internal controls for quantitative RT-PCR (qRT-PCR). The stability of expression of seven putative reference genes (*Actb*, *B2m*, *Gapdh*, *Hprt1*, *Mapk1*, *Ppia* and *Rn18s*) was analyzed using geNorm, BestKeeper and Normfinder software. In addition, several housekeeping genes were tested as loading controls for Western blot in the same sample panel, using Image J. Overall, for qRT-PCR the combination of *Gapdh* and *Mapk1* showed the highest stability for most experimental sets. *Actb* was downregulated in more mature stages, while *Rn18s* and *Hprt1* showed the highest variability. We normalized the expression of cyclin D1 using various reference genes and demonstrated that spurious results may result from blind selection of internal controls. For Western blot significant variation could be seen among four putative internal controls (β-actin, cyclophilin b, α-tubulin and lamin A/C), while MAPK1 was stably expressed.

**Conclusion:**

Putative housekeeping genes exhibit significant variation in both mRNA and protein content during retinal development. Our results showed that distinct combinations of internal controls fit for each experimental set in the case of qRT-PCR and that MAPK1 is a reliable loading control for Western blot. The results indicate that biased study outcomes may follow the use of reference genes without prior validation for qRT-PCR and Western blot.

## Introduction

Gene expression analyses are crucial for the discovery and characterization of the roles for known genes [1]. Concerning the study of the development of different tissues, these analyses can provide insights into complex regulatory networks that coordinate proliferation, cell commitment, differentiation and apoptosis. Considering the complexity of the neural tissue a major issue is the standardization of quantitative approaches to investigate expression patterns in response to specific treatments or throughout development. In this study, we addressed this question focusing on retinal development.

The retina is derived from the diencephalon, and is responsible for the conversion of electromagnetic energy into nerve impulses [2]. Vertebrate retinas are composed of seven major cell types that are produced from multipotent progenitor cells [3], [4]. During development, these progenitors expand through cell proliferation, commit to distinct cell types and exit the cell cycle to generate either retinal neurons or the Müller glia in an evolutionary conserved birth order [5]. Two types of photoreceptors are responsible for phototransduction and while cones are involved in photopic and color vision, rods are responsible for scotopic vision [2]. Photoreceptors signal to bipolar cells and these to ganglion cells, while information is laterally processed through horizontal and amacrine cells [2]. Retinal ganglion cells carry the visual input to the brain through the optic nerve [2] .

Quantitative real-time reverse transcription–polymerase chain reaction (qRT-PCR) and Western blot are widely used to quantify RNA and protein content, respectively. qRT-PCR is highly sensitive, allowing the quantification of rare transcripts. It has high specificity, good reproducibility, and a wide dynamic range [6], [7]. Western blot is a semi-quantitative method used to identify individual proteins in complex protein extracts. It has high specificity due to antigen-antibody interaction, which can be verified by checking the expected molecular weight. Signal amplification is obtained through the use of primary and secondary antibodies [8]. Experimental errors can, however, be introduced at multiple steps in both protocols due to variability in the amount of starting material, extraction and pipetting [9]. In the case of RNA, additional variation is found owing to the efficiency of reverse transcription or the amount of input template, whereas transfer efficiency is a recurrent problem in Western blot. So, it is essential to account for experimental variance as well as biological differences when conducting gene expression studies.

The ratio between target and internal control is used to standardize independent biological samples [7]. The stability of expression of an internal or loading control is required for accurate and reliable normalization in both qRT-PCR experiments and Western blot [7], [10]. The reference genes are typically selected because of their role in key biological pathways, ubiquitous expression and similar expression levels among all samples. However, several so-called housekeeping genes commonly used as reference can be dynamically expressed either in response to treatments or throughout development [11], [12].

It is likely that many constitutive genes are not stable during retinal development. For example, expression of both β-actin (*Actb*) and glyceraldehyde-3-phosphate-dehydrogenase (*Gapdh*) were shown to vary with tissue maturation [13], [14], [15], [16], [17], [18], [19]. Indeed, a proteomics study of the developing mouse retina identified a large set of proteins whose expression is altered throughout development, including β-actin and tubulin α-1 chain [12]. Therefore, selection of up- or downregulated genes as reference during retinal maturation may affect statistical parameters such as power and sample size [20], [21]. Proper verification of suitable endogenous controls would prevent inadequate quantification and spurious findings. This is particularly relevant in the case of genes that suffer subtle changes throughout development or under experimental conditions.

Many studies have been carried out on animal and plant samples to describe reference genes for normalization [1], [10], [11], [22], [23], [24]. Several tools have been developed to identify the most stably expressed genes in a specific setup, but none is universally accepted. Here, we report the validation of suitable internal controls for expression analysis by qRT-PCR during development of the rat retina, with the use of geNorm [1], BestKeeper [9] and NormFinder [25] software. The seven genes tested in this study were chosen based on its previous use as reference genes [26], [27], [28], [29], [30] and also because of its involvement in diverse cellular processes, which reduces the probability of co-regulation.

This is the first in-depth study to validate internal controls for expression analysis throughout retinal development. The relevance of this evaluation was illustrated by changing results of cyclin D1 RNA measurements when inappropriate reference genes were used for normalization. In addition, we validated loading controls for Western blot analysis across the same sample panel.

## Results

### Validation of loading control for Western blot

**Figure 1:**
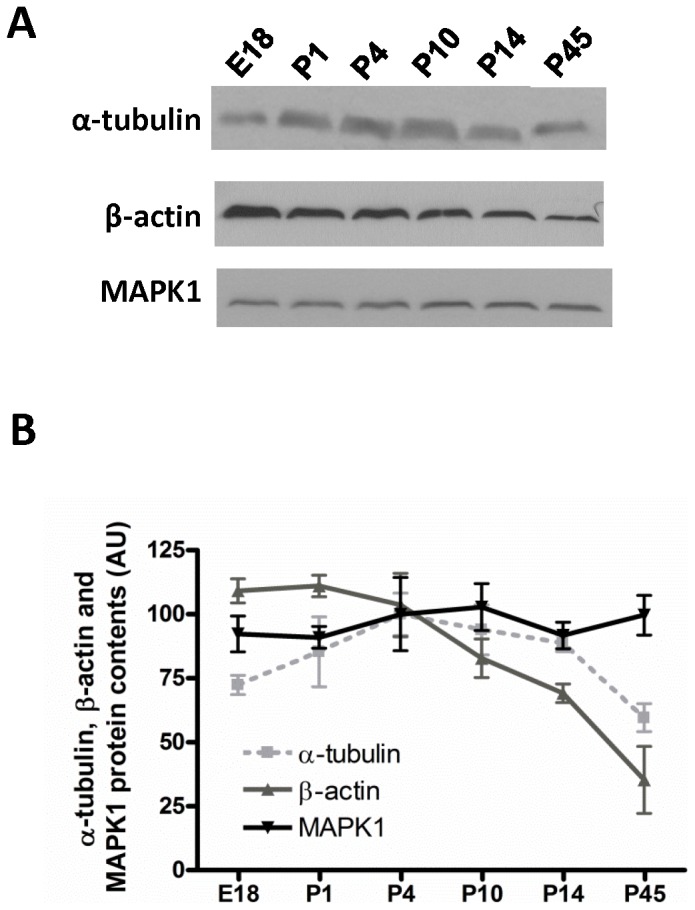
Validation of loading controls for Western blot throughout retinal development. A representative Western blot of α-tubulin, β-actin and MAPK1 is shown in A for distinct retinal developmental stages (E18, P1, P4, P10, P14, P45). (B) Densitometric analysis of β-actin, α-tubulin and MAPK1. Results are presented as means ± SEM pooled from three independent experiments.

To identify an internal control with stable expression throughout retinal development, we characterized the protein content of two commonly used housekeeping genes, β-actin (ACTB) and α-tubulin; MAPK1 (ERK2), previously used as loading control by our group [27], [31]; Cyclophilin B and Lamin A/C. Lamin A/C and Cyclophilin B were not detected in all stages of development (data not shown). Protein extracts were sampled at least three times from each developmental stage from embryonic (E) to post-natal (P) days: E18, P1, P4, P10, P14 and P45 (Figure 1A). Furthermore, to analyze the variation of expression among the three candidates detected in all stages, immunoblot was performed in the same membrane for each biological replicate after stripping, so that loading errors would not mask the results.

β-actin showed an evident variation during development with a Coefficient of variation (Cv) of 36.6%. β-actin protein content at E18 was significantly higher than at P45 (109.1±4.8 in E18 versus 35.3±13.1 in P45, p<0.001). Significant differences were detected also when P1 was compared to P14 and P45 (P1 111.0±4.2 versus P14 69.11.4±3.6, p<0.05; P45 35.3±13.1, p<0.001). Expression level at P4 was significantly higher than P45 (P4 103.6±12.39 versus P45 35.3±13.1, p<0.001), and expression at P10 was also significantly higher than at P45 (P10 82.8±7.5 versus P45 35.3±13.1, p<0.05) (Figure 1B).

In contrast, α-tubulin showed less variation with a Cv of 24.5%, but could be used properly as an internal control only if there is no intent to evaluate the expression at mature stages. This is because the content of α-tubulin at P45 was significantly different than at P4 (P45 59.6±5.5 versus P4 99.9±8.3, p<0.05). Although a tendency is also observed when E18 is compared to P4 and P10, the difference was not statistically significant. Similarly, this is observed when P45 is compared to P10. (Figure 1B).

MAPK1 protein content was slightly variable between the ages analyzed and the Cv was approximately two-fold lower than β-actin, 18.5% (Figure 1B). Thus, MAPK1 is, among the proteins tested, the most reliable loading control, although this gene is not commonly recognized as a housekeeping gene [11].

### Selection of reference genes for qRT-PCR

Two samples at each of various stages of retinal maturation were analyzed and combined into five experimental sets: embryonic to early postnatal retina (Group 1- E18, P1 and P4), proliferative to non-proliferative transition (Group 2 - P1, P4 and P10), early postnatal to mature retina (Group 3- P1, P4, P10, P14, P45), Late postnatal to mature retina (Group 4- P10, P14 and P45) and embryonic to mature retina (Group 5- all ages: E18, P1, P4, P10, P14, P45).

**Figure 2:**
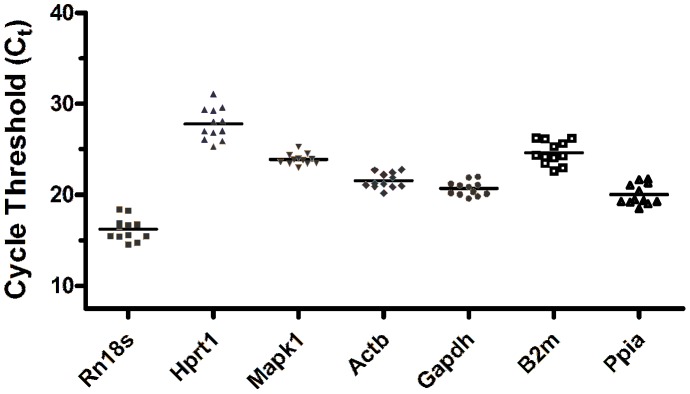
Ct distribution of each putative reference gene among samples. Cycle threshold (Ct) was determined for each reference gene tested and its distribution from 12 samples obtained from 6 distinct retinal developmental stages was determined (E18, P1, P4, P10, P14 and P45, 2 samples for each stage).

For each sample, qRT-PCR was done for seven candidate reference genes (Table S1) with technical triplicates. For each of the two biological replicates, RNA was extracted from 2–3 pooled individuals, so that the RNA could be considered an average sample of the developmental stage analyzed. The distribution of Ct data among the 12 samples is shown in Figure 2.

**Figure 3:**
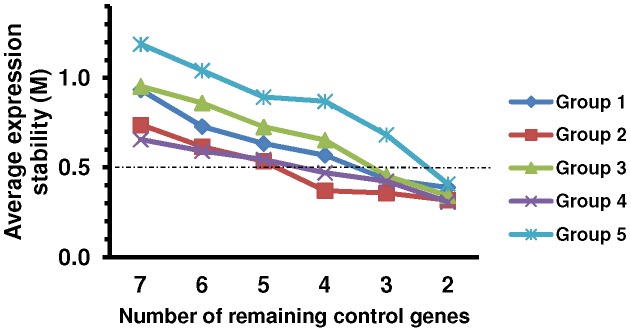
Average expression stability calculated by stepwise exclusion with geNorm for the reference genes. Average expression stability values were obtained for each experimental set (Groups 1–5). Pairwise variation decreases from left to right, due to stepwise exclusion of the least stable reference gene. M values below the theoretical threshold of 0.5 indicate adequate gene stability. The corresponding reference genes are ranked in Table 1. Group 1: E18, P1 and P4; Group 2: P1, P4 and P10; Group 3: P1, P4, P10, P14, P45; Group 4: P10, P14 and P45; Group 5: all ages.

**Figure 4:**
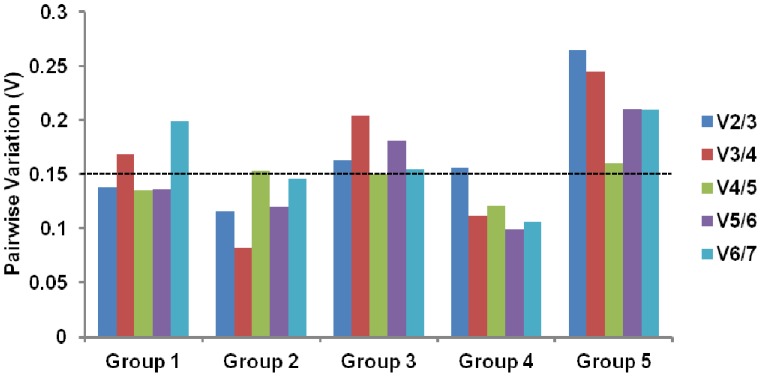
Pairwise variation analysis among sequential normalization factors calculated by geNorm. Determination of the optimal number of control genes by calculation of the pairwise variation coefficient. A value from 0.15 to 0.20 was determined as an appropriate cut-off. To the right in each group, the increasing values are due to the inclusion of unstable reference genes. Group 1: E18, P1 and P4; Group 2: P1, P4 and P10; Group 3: P1, P4, P10, P14, P45; Group 4: P10, P14 and P45; Group 5: all ages.

For geNorm use, relative values calculated by 2^-ΔCT^ method were imported and the analysis provided a measure of gene expression stability (M) for each reference gene from least (highest M value) to most stable (lowest M value) (Figure 3; Table 1). geNorm also generated other two outcomes, the pairwise variation value V_n/n+1_ and the effects of step wise inclusion of the next most stable reference gene on V_n/n+1_ (Figure 4).

**Table 1:** Reference genes ranked by their expression stability (M) calculated by geNorm for different combination of samples.

Group 1		Group 2		Group 3		Group 4		Group 5	
Ranking	Stability value (M)	Ranking	Stability value (M)	Ranking	Stability value (M)	Ranking	Stability value (M)	Ranking	Stability value (M)
*Gapdh*	0,389	*Actb*	0,318	*Gapdh*	0,343	*Gapdh*	0,308	*Mapk1*	0,409
*B2m*	0,389	*Mapk1*	0,318	*Mapk1*	0,343	*Rn18s*	0,308	*Gapdh*	0,409
*Mapk1*	0,435	*Rn18s*	0,358	*Rn18s*	0,455	*Mapk1*	0,424	*Rn18s*	0,683
*Actb*	0,569	*Gapdh*	0,371	*Actb*	0,653	*Ppia*	0,471	*Actb*	0,869
*Ppia*	0,634	*B2m*	0,535	*Ppia*	0,727	*Hprt1*	0,545	*Ppia*	0,893
*Rn18s*	0,729	*Hprt1*	0,616	*Hprt1*	0,86	*B2m*	0,592	*B2m*	1,041
*Hprt1*	0,934	*Ppia*	0,738	*B2m*	0,953	*Actb*	0,656	*Hprt1*	1,188

The reference genes are ranked using the expression stability value (M) obtained from the geNorm analysis. Stability decreases from top to bottom. Group 1: E18, P1 and P4; group 2: P1, P4 and P10; group 3: P1, P4, P10, P14, P45; group 4: P10, P14 and P45; group 5: all ages.

First, we analyzed the expression stability for Group 1 (E18, P1 and P4). *Gapdh*, *B2m* and *Mapk1* showed M values below the theoretical threshold of 0.5, indicating adequate gene stability (Figure 3; Table 1). M value increased moderately for *Actb*, whilst *Ppia*, *Rn18s* and *Hprt1* showed higher variability with an increased slope of M value curve. Importantly, *Rn18s*, which is commonly used as an internal control, showed the second largest M value. The optimal number of reference genes for use in standardization can be deduced from pairwise variation. A value from 0.15 to 0.20 is generally considered as an appropriate cutoff for the pairwise variation, although this should be regarded as a reference rather than a strict value [1], [32], [33]. Below this threshold the addition of an extra reference may not result in significant improvement, and even lead to reduced average expression stability. In Group 1, the pairwise variation V_2/3_ yielded a value of 0.138, which was already below 0.15 (Figure 4). This means that, based on geNorm, a combination of *Gapdh* and *B2m* is stable enough to standardize Group 1, without the need to add *Mapk1*. The stability of the candidates was further analyzed by BestKeeper and Normfinder software. The coefficient of variance calculated by BestKeeper (Table 2) based on Ct values of Group 1 samples led to the same ranking as geNorm (Table 1). On the other hand, Normfinder, which takes into account both intra- and intergroup variations for calculation of the normalization factor, pointed at *Mapk1* and *B2m* as the best combination of reference genes (Table 3).

**Table 2:** Bestkeeper ranking for each group of samples with mean ± SD (standard deviation) and coefficient of variance (CV) of threshold cycle (Ct) values.

Group 1			Group 2			Group 3			Group 4			Group 5		
Ranking	Cq	CV%	Ranking	Cq	CV%	Ranking	Cq	CV%	Ranking	Cq	CV%	Ranking	Cq	CV%
*Gapdh*	21.11±0.48	1.98	*Actb*	21.26±0.39	1.84	*Mapk1*	23.78±0.43	1.80	*Mapk1*	23.51±0.25	1.07	*Mapk1*	23.85±0.44	1.83
*B2m*	25.62±0.56	2.19	*Mapk1*	24.00±0.52	2.15	*Gapdh*	20.49±0.56	2.75	*Gapdh*	20.22±0.32	1.56	*Gapdh*	20.67±0.65	3.13
*Mapk1*	24.20±0.50	2.35	*Gapdh*	20.56±0.68	3.29	*Actb*	21.71±0.68	3.14	*B2m*	23.56±0.58	2.47	*Actb*	21.51±0.73	3.39
*Actb*	21.08±0.56	2.63	*B2m*	24.94±1.02	4.10	*B2m*	24.36±0.97	3.96	*Actb*	21.95±0.59	2.70	*B2m*	24.59±1.08	4.38
*Ppia*	19.52±0.69	3.52	*Ppia*	19.85±0.91	4.61	*Hprt1*	27.29±1.16	4.26	*Hprt1*	26.66±0.73	2.73	*Ppia*	20.01±0.94	4.70
*Rn18s*	16.23±1.23	4.26	*Hprt1*	27.64±1.33	4.80	*Ppia*	20.18±0.96	4.74	*Ppia*	20.51±0.73	3.58	*Hprt1*	27.77±1.44	5.20
*Hprt1*	28.88±1.07	6.58	*Rn18s*	16.13±1.21	7.47	*Rn18s*	16.29±1.13	6.92	*Rn18s*	16.25±1.00	6.13	*Rn18s*	16.24±1.03	6.36

CV: coefficient of variance is expressed as the percentage of Ct standard deviation to the mean Ct. Stability decreases from top to bottom. Group 1: E18, P1 and P4; group 2: P1, P4 and P10; group 3: P1, P4, P10, P14, P45; group 4: P10, P14 and P45; group 5: all ages.

**Table 3:** NormFinder analysis with reference genes ranked by their expression stability or different experimental sets.

Group 1		Group 2		Group 3		Group 4		Group 5	
Ranking	Stability value	Ranking	Stability value	Ranking	Stability value	Ranking	Stability value	Ranking	Stability value
*Mapk1*	0.208	*Gapdh*	0.219	*Gapdh*	0.272	*Gapdh*	0.217	*Gapdh*	0.305
*B2m*	0.264	*Mapk1*	0.244	*Mapk1*	0.403	*Ppia*	0.297	*Mapk1*	0.387
*Gapdh*	0.274	*B2m*	0.441	*Hprt1*	0.528	*Hprt1*	0.302	*Rn18s*	0.593
*Rn18s*	0.476	*Actb*	0.498	*Rn18s*	0.568	*B2m*	0.307	*B2m*	0.623
*Actb*	0.524	*Rn18s*	0.528	*Ppia*	0.582	*Mapk1*	0.394	*Ppia*	0.713
*Ppia*	0.531	*Ppia*	0.564	*B2m*	0.593	*Actb*	0.406	*Actb*	0.771
*Hprt1*	0.692	*Hprt1*	0.584	*Actb*	0.666	*Rn18s*	0.436	*Hprt1*	0.802

Best combination of two genes	*Mapk1* and *B2m*	Best combination of two genes	*Gapdh* and *Mapk1*	Best combination of two genes	*Gapdh* and *Mapk1*	Best combination of two genes	*Gapdh* and *Ppia*	Best combination of two genes	*Gapdh* and *Mapk1*
**Stability value**	0.182	**Stability value**	0.149	**Stability value**	0.263	**Stability value**	0.194	**Stability value**	0.272

The reference genes are ranked using the expression stability value obtained from the NormFinder analysis. Stability decreases from top to bottom. Group 1: E18, P1 and P4; group 2: P1, P4 and P10; group 3: P1, P4, P10, P14, P45; group 4: P10, P14 and P45; group 5: all ages.

For Group 2 (P1, P4 and P10), *Actb*, *Mapk1*, *Rn18s* and *Gapdh* had M values below the theoretical threshold of 0.5 (Figure 3). M value for *B2m* is 0.535 so it was not a good candidate for Group 2 normalization. These data reinforce that each experimental setup may have its best combination of reference genes. Pairwise variation V_2/3_ achieved a value of 0.116 (Figure 4). Therefore, based on geNorm, *Actb* and *Mapk1* are stable enough to standardize Group 2. BestKeeper outcome showed that *Actb* and *Mapk1* had the lowest coefficient of variance (Table 2). On the other hand, Normfinder pointed at *Gapdh* and *Mapk1* as the best combination of reference genes (Table 3).

*Gapdh*, *Mapk1* and *Rn18s* showed M values below the theoretical threshold of 0.5 for Group 3 (P1, P4, P10, P14, P45) (Figure 3). Pairwise variation V_2/3_ had a value of 0.163 (Figure 4). Thus, geNorm would recommend the use of the two most stable genes *Gapdh* and *Mapk1*. In addition, Bestkeeper and Normfinder confirmed the stability of both genes (Table 2 and Table 3).

Analysis of Group 4 (P10, P14 and P45) showed discrepancies among the softwares. geNorm picked *Gapdh* and *Rn18s* as the most stable reference genes (Figure 3; Table 1). Moreover, *Mapk1* and *Ppia* showed M values below 0.5 and Pairwise variation V_2/3_ had a value of 0.156 (Figure 3 and 4). Bestkeeper calculated the lowest coefficient of variance for *Mapk1* and *Gapdh*, while Normfinder chose *Gapdh* and *Ppia* as most stable genes (Table 2 and Table 3). It is important to note that *Rn18s* and *Ppia* had the highest coefficients of variance (Table 2).

The same analysis was performed for all samples together (Group 5- all ages: E18, P1, P4, P10, P14, P45), so that we could compare the expression levels of target genes within a broader range of ages. *Gapdh* and *Mapk1* were ranked by all three software as the most stable genes to normalize Group 5 (Figure 3; Tables 1, 2, 3), but only V_4/5_ geNorm pairwise variation achieved an appropriate value of 0.16 (Figure 4). Therefore, although an agreement on the two more stable genes were observed for all programs, based on geNorm the use of the four most stable genes would be recommended.

**Figure 5:**
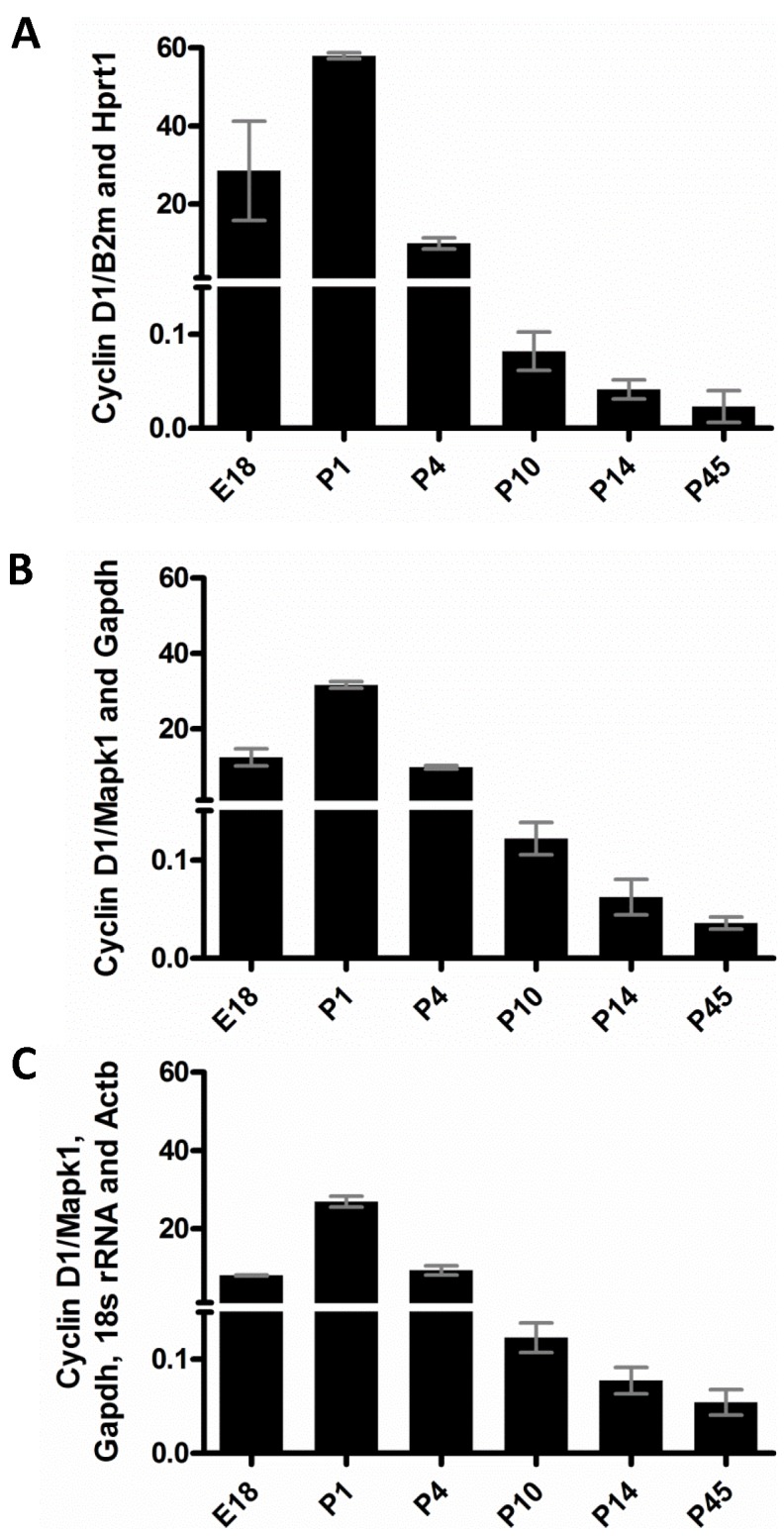
Effect of different choices of reference genes on the analysis of Cyclin D1. Cyclin D1 mRNA content from samples of all retinal stages tested was normalized with *Hprt1* and *B2m* (A), *Mapk1* and *Gapdh* (B) *Mapk1*, *Gapdh*, *Rn18s* and *Actb*. The results of comparative Ct (ΔΔCt) method are represented as means ± SD for Group 5 (E18, P1, P4, P10, P14 and P45).

Finally, we assessed the effect of a blind selection of reference genes on the normalization of cyclin D1 expression. Using Group 5, which contains all ages tested, normalization against the most variable candidates (*B2m* and *Hprt1*) led to a dramatic increase in standard deviation and discrepancies in cyclin D1 expression pattern (Figure 5A) when compared to the normalization with the 4 most stable genes, as recommended by geNorm (Figure 5B) or with *Mapk1* and *Gadph*, which were indicated as the most stable genes by geNorm, Normfinder and Bestkeeper (Figure 5C). These last two conditions presented very similar results (Figures 5B and 5C). Our results led us to recommend the internal controls indicated in Table 4 (see details in Discussion).

**Table 4:** Adequate internal controls for different combinations of retinal developmental stages.

Experimental sets				
Group 1	Group 2	Group 3	Group 4	Group 5
*Mapk1* and *B2m*	*Gapdh* and *Mapk1*	*Gapdh* and *Mapk1*	*Gapdh* and *Ppia*	*Gapdh* and *Mapk1*

Internal controls for each group were elected with criteria described in Discussion.

Group 1: E18, P1 and P4; group 2: P1, P4 and P10; group 3: P1, P4, P10, P14, P45; group 4: P10, P14 and P45; group 5: all ages.

## Discussion

The ideal control gene should have similar expression regardless of experimental conditions, such as: developmental stages, composition of cell types, and/or sample treatments. This applies both for Western blot and qRT-PCR. Indeed, this problem has been addressed for gene expression studies using qRT-PCR [1], [22], [23], [24], but few studies have already characterized reliable loading controls for Western blot [10]. Adequate selection of reference genes is critical for sensitive and accurate quantification of mRNA or protein content, especially for those genes whose transcript and protein levels are low.

In the present study, we demonstrated a significant decrease in β-actin protein content along retinal development, although it is a housekeeping gene frequently used as an internal control [26], [34], [35] (Figure 1A, B). α-tubulin showed a coefficient of variance lower than β-actin, but a significant difference was still found between P45 and P4, and a tendency was identified when E18 is compared to P4 and P10 although the difference was not statistically significant (Figure 1B). For standardization of a broader range of developmental ages from embryonic to mature retina, MAPK1 was the most stable choice (Figure 1), as empirically observed in our laboratory [27]. In spite of the lack of prior evidence on constitutive expression of MAPK1, there were not significant differences in MAPK1 protein content throughout retinal development. In conclusion, our findings highlight a critical problem in previous investigations that used β-actin for normalization, and may be helpful for further studies on retinal development.

Using geNorm, BestKeeper and Normfinder algorithms, we were able to find the best combination of reference genes for qRT-PCR of various groups of samples. NormFinder is an algorithm for the identification of the optimal pair of reference genes out of a group of candidates. This software uses information about expression stability, as well as the variation between sample subgroups to examine each gene independently and test combinations of gene pairs to compensate the variability of the system [25]. geNorm selects the best two internal control genes with similar intergroup variation [1], whereas BestKeeper calculates the coefficient of variance of each putative reference gene, which is defined as a percentage of the average Cycle threshold (Ct) level [9]. The minimum number of endogenous control required for normalization in gene expression studies is a major aspect in debate. Hence, it is worth considering a balance between the absolute gain in statistical power and the extra cost and effort when using additional reference genes.

In this study, NormFinder was the software considered as the most reliable choice because it takes into account both intra- and inter-group variations for normalization. However, geNorm and BestKeeper were also relevant to elect internal controls for each experimental set. Normfinder pointed *Mapk1*/*B2m* and *Gapdh*/*Mapk1* as the best combination to normalize Group1 and Group 2, respectively (Table 4). In both cases, the two reference genes recommended by Normfinder were top ranked by the other programs. *Gapdh* and *Mapk1* were selected for Group 3 normalization by all software and showed a geNorm Pairwise variation V_2/3_ value of 0.163. As Pairwise M cutoff values of 0.20 to 0.15 are suggested [1], [32], [33], we conclude that there is no need to add a third reference gene (Table 4). geNorm, Normfinder and BestKeeper showed divergent results for Group 4, as expected because they are based on distinct statistical algorithms. Our conclusion is that *Gapdh*/*Ppia* would work well to normalize Group 4 target expression based on Normfinder algorithm. One can argue that *Ppia* and *Rn18s* showed the highest coefficients of variance, but it is important to notice that Bestkeeper software does not take into account the stability of a combination of genes. Due to the discrepancies mentioned above and the higher reliability of Normfinder algorithm as stated above, we advise the use of *Gapdh*/*Ppia*.

The more complex is the experimental set, harder it becomes to find stably expressed genes. When the same 7 genes were tested for Group 5, which includes all ages tested, *Gapdh* and *Mapk1* were ranked by all three software as the two more stable reference genes for normalization (Figure 3, Table 1, 2, 3). When geNorm is applied, the use of the four most stable genes is recommended, since the Pairwise variation V_4/5_ value was 0.16 (Figure 4). *Mapk1* and *Gapdh* proved to be reliable internal controls for normalization of Group 5, as low sample variance and the same pattern of expression was obtained when they were used to analyze the expression of cyclin D1 (Figure 5B), compared to the use of the combination of the four reference genes indicated by geNorm (Figure 5C). *Mapk1* and *Gapdh* showed robust constitutive expression throughout retinal development. *Mapk1* presented a coefficient of variation 30% lower than, for example, the most stable gene investigated in mouse myocardial infarction models [21]. Alternatively, if there is no intention to include the analysis of the embryonic stage, the combination of *Gapdh* and *Ppia* would be the most reliable choice.

When searching for reference genes commonly used to compare different stages of retinal development in rodents, it is common to find the use of only one [26], [36], [37], or at most two reference genes [28], [38] for qRT-PCR. The most frequent reference genes used are *Actb* or *Gapdh*, but *Rn18s*, *Hprt1* and *Prkcα* are also described [26], [28], [36], [37], [39]. Consistent with our data, a single cell study observed an expressive variation on the expression of housekeeping genes among progenitor cells isolated from different stages of retinal development [40].

In conclusion, we demonstrated for the first time how relevant it is to validate a reference gene set suitable for expression studies on rat retinal development. Our results indicate combinations of genes for qRT-PCR analysis for different combinations of developmental stages and MAPK1 as the loading control for Western blot.

We furthermore advise against the use of *β-actin* for both methods particularly when a long range of developmental stages are analyzed (exemplified by Group 5 in this study, which range from E18 to P45), because this gene is downregulated during retinal development. Given the risk of substantial variation on gene expression among distinct animal models, we encourage the validation of reference genes as an initial and essential step in quantitative studies of either mRNA (qRT-PCR) or protein content (Western blot). This is particularly relevant when comparing tissue extracts from various stages of development, due to the variety of cellular processes modulated throughout morpho- and histogenesis.

## Materials and Methods

### Materials

Dulbecco's Modified Eagle Medium (DMEM), UltraPure DNase/RNase-Free water and Trizol were purchased from Invitrogen (Calsbad, CA, USA). First-strand cDNA synthesis kit was purchased from GE Healthcare (Little Chalfont, UK). DNA-free kit and QuantumRNA™ rRNA 18S Internal Standards Kit from Ambion (Austin, TX, USA), Power SYBR Green PCR Master Mix and optical 96-well plates from Applied Biosystems (Foster City, CA, USA) were used. Primers were purchased from Integrated DNA Technologies, USA. Luminata™ Forte Western HRP Substrate was purchased from Millipore (Billerica, MA, USA). Secondary antibodies linked to horseradish peroxidase (HRP) were from Cell Signaling (Beverly, MA, USA). All information about the primary antibodies used is described in Table S2.

### Samples

All experimental procedures with animals for this study were approved by the Ethics Committee on Animal Experimentation of the Health Sciences Center of the Universidade Federal do Rio de Janeiro (CEUA/CCS/UFRJ) under the protocol number IBCCF121, based on currently accepted international rules. Retinas were dissected in DMEM from the eyes of Lister hooded rats from various stages (E18, P1, P4, P10, P14 and adult). Embryos were removed from the uterus of pregnant rats euthanized in a carbon dioxide chamber. While the same procedure was performed to kill adult rats, pups and embryos were killed by instantaneous decapitation. Each of the two biological replicates of RNA was extracted from 2–3 pooled individuals. Therefore, the RNA obtained from each biological replicate could be considered an average sample of the developmental stage analyzed [23], [41], [42], [43], [44], [45]. For western blot analysis at least three independent biological replicates were used.

### mRNA extraction and cDNA synthesis

Each pool of retinas of rats at the ages E18, P1, P4, P10, P14 and P45 was washed once with PBS and RNA was extracted with Trizol following manufacturer's instructions. RNA integrity was confirmed by visualization of RNA 18S and 28S in 1% agarose gel electrophoresis. RNA was treated with DNA-free kit following manufacturer's instructions, quantified with NanoDropTM Spectrophotometer ND-1000 (Thermo Scientific) and stored at −80°C. Quantity and quality of RNA extracted were assessed to confirm good RNA yields and purity with a mean A260/A280 ratio of 1.9±0.2. DNA contamination was ruled out by standard PCR and agarose gel electrophoresis. cDNA was synthesized from 1 µg of RNA with pd(N)6 random primers, as described in kit manual (First Strand cDNA Synthesis Kit, Amersham).

### Primer Design, specificity and efficiency

Primers for qRT-PCR were designed with Primer Quest (Integrated DNA Technologies SciTools) with the following criteria: product size ranging from 80 to 285 bp, optimum Tm of 60°C and GC content about 50%. Secondary structures and primer-dimers were avoided. *Rn18s* primers were from Ambion (Austin, TX, USA). Standard RT-PCR confirmed that the primers amplified only a single product with expected size (data not shown). Primer efficiency was calculated for qRT-PCR using the slope of the calibration curve according to the equation: E = 10 [−1/slope] [7]. All information about the primers was included in Table S1.

### Quantitative RT-PCR (qRT-PCR)

For qRT-PCR reactions were carried out in an optical 96-well plate in ABI7500 (Applied Biosystems). The primers used for quantitative PCR analysis are listed in Table S1. Control without reverse transcriptase was performed to ensure that the results were not due to amplifications of genomic DNA. PCR product identity was confirmed by melting-point analysis. Each reaction contained 12.5 µL of SYBR Green 2× reaction mix, 2 µL of diluted cDNA (1∶65), 100 nM of each primer (0.5 µL each) and 9.5 µL of UltraPure DNase/RNase-Free water (Invitrogen). Conditions used were: 50°C/2 min; 95°C/10 min and 45 amplification cycles of 95°C/15 s; 60°C/60 s. In order to reduce confounding variance, two independent biological samples from different littermates were analyzed in technical triplicates. Technical replicates were averaged before all software analysis.

### Reference gene expression stability and statistical analysis

Expression levels of seven putative housekeeping genes in all the samples were determined in the exponential phase by the number of cycles necessary to reach the prior established threshold (Ct) (Table S1). The Ct values were converted to a linear scale by the equation: 2^−ΔCT^ [46]. These data were used as the input to verify the expression stability at geNorm v3.5, BestKeeper and Normfinder tools, following instructions [1], [9], [25]. geNorm algorithm is based on the geometric averaging of multiple control genes and mean pairwise variation of a gene from all other control genes in a given set of samples. This algorithm also calculates the pairwise variation value, which indicates the optimal number of control genes to be used, and cutoff values of 0.20 [33] to 0.15 [1], [32] have been suggested. NormFinder takes into account intra- and intergroup variations for normalization factor (NF) calculations [25]. The expression values of Cyclin D1 were assessed to test the efficacy of the selected endogenous controls. The comparative Ct method (ΔΔCt) was used to determine the target quantity in sample as compared with the mean of different reference genes in combination and relative to a calibrator [(Ct_target gene_ - Ct_reference gene_)_sample_ - (Ct_target gene_ - Ct_reference gene_)_calibrator_]. A similar mathematical correction similar to the one of the software qBase, which is based on the use of the average of ΔCt of all groups (in this case: E18, P1, P4, P10, P14 and P45), was applied to define the calibrator [47].

### Western blots

Dissected retinas from 3 different littermates for each stage were washed with PBS and total protein was extracted (10 mM Tris base- HCl, 150 mM NaCl, 1% NP-40, 1% Triton X-100, 5 mM EDTA, 0.1% SDS, 1% sodium deoxicholate, 1 mM phenylmethylsulfonyl fluoride, 1 µg/ml aprotinin, 1 µg/ml pepstatin, 1 µg/ml leupeptin, 1% sodium orthonovadate and 50 mM sodium fluoride). The concentration of lysates was determined by the Lowry assay [48]. Lysates (30 µg) were separated in SDS-polyacrylamide gels and transferred to nitrocellulose membranes. Membranes were blocked with 5% non-fat dry milk and incubated with primary antibodies to five putative constitutively expressed proteins, listed in Table S2, followed by horseradish peroxidase-conjugated secondary antibodies. Immunoblots were developed with Luminata (Millipore), according to manufacturer's instructions, and densitometric results were analyzed with Image J software. Coefficients of variance were calculated by the ratio between standard deviation and mean. Stripping was performed by incubation with glycine 2M pH 2.2 for 20 minutes at room temperature.

### Statistical analyses

For Western blot, statistical comparisons between more than two experimental groups were made with one-way anova tests followed by Bonferoni multiple comparisons test. Results are reported as mean ± standard error of the mean (SEM), and p was set to 0.05. For all analyses, prism 4.0 software (GraphPad Software, San Diego, CA, USA) was used.

## Supporting Information

Table S1 Primer sequences for seven putative endogenous control genes and target gene. Detailed description of all genes tested, primer pairs' sequences, PCR conditions and primers efficiencies.
(TIF)

Table S2 Antibodies against five putative internal controls for Western Blot. Detailed description of all protein tested, antibodies and conditions used for Western Blot analyses.
(TIF)
