# Supplementary figures and images for: Avoiding Pitfalls of Internal Controls: Validation of Reference Genes for Analysis by qRT-PCR and Western Blot throughout Rat Retinal Development
Source: PLoS One. 2012 Aug 20;7(8):e43028. doi: 10.1371/journal.pone.0043028 (PMC3423434; doi:10.1371/journal.pone.0043028)

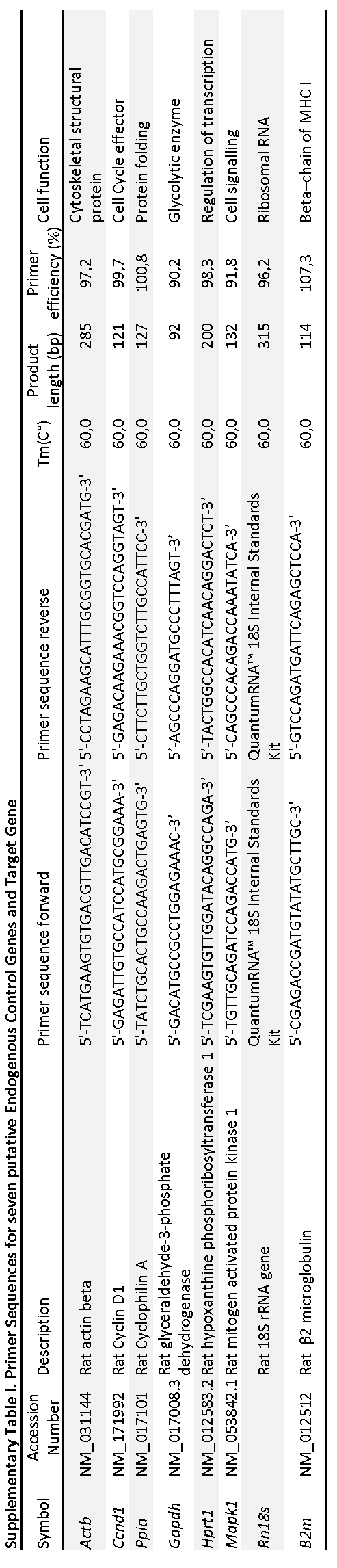

Supplement: Table S1 — Primer sequences for seven putative endogenous control genes and target gene. Detailed description of all genes tested, primer pairs' sequences, PCR conditions and primers efficiencies. (TIF) [file pone.0043028.s001.tif]

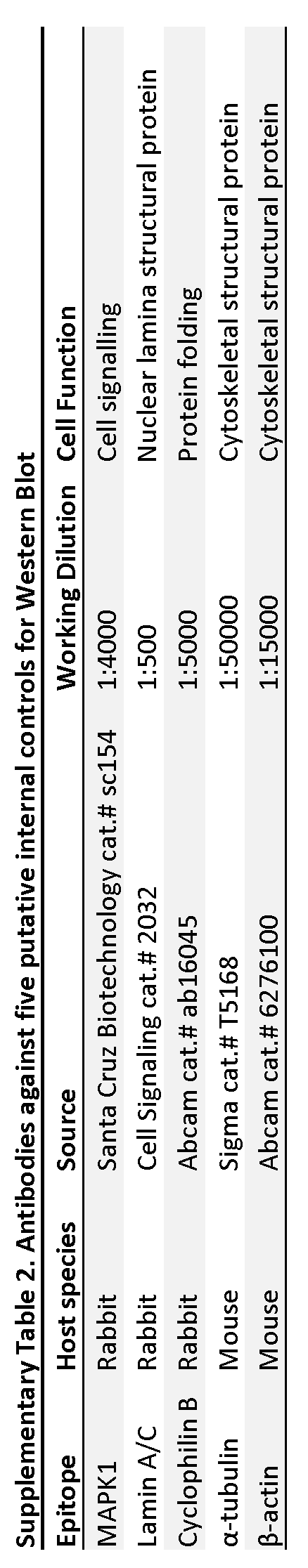

Supplement: Table S2 — Antibodies against five putative internal controls for Western Blot. Detailed description of all protein tested, antibodies and conditions used for Western Blot analyses. (TIF) [file pone.0043028.s002.tif]
